# Serum Metabolomics Revealed the Differential Metabolic Pathway in Calves with Severe Clinical Diarrhea Symptoms

**DOI:** 10.3390/ani10050769

**Published:** 2020-04-28

**Authors:** Mei-Zhou Huang, Dong-An Cui, Xiao-Hu Wu, Wang Hui, Zuo-Ting Yan, Xue-Zhi Ding, Sheng-Yi Wang

**Affiliations:** 1 Lanzhou Institute of Husbandry and Pharmaceutical Sciences of Chinese Academy of Agricultural Science, Lanzhou 730050, China; 13141254071@163.com (M.-Z.H.); dogan@126.com (D.-A.C.); wuxiaohu01@caas.cn (X.-H.W.); wanghui01@caas.cn (W.H.); yanzuoting@caas.cn (Z.-T.Y.); 2 Key Laboratory of Veterinary Pharmaceutical Development of Ministry of Agriculture, Engineering and Technology Research Center of Traditional Chinese Veterinary Medicine of Gansu Province, Key Laboratory of New Animal Drug Project of Gansu Province, Lanzhou 730050, China

**Keywords:** diarrhea, calf, metabolomics, liquid chromatography–mass spectrometry, serum

## Abstract

**Simple Summary:**

The present study focuses on the metabolic changes in the diarrhea of calves, which are manifested with the following symptoms: a thin water-like stool, cold ears and nose, throbbing bowels, oliguria, a pale or yellowish complexion, a smooth mouth, and a slow pulse. The differential metabolic pathways in calves with diarrhea were screened by metabolomics. There were nine biomarkers in the serum of healthy calves and calves with diarrhea. On the basis of these biomarkers, their associated mineral absorption, protein digestion and absorption, and other metabolic pathways, the targeted regulation of the metabolic differences of calves with diarrhea may contribute to the diagnosis, treatment, and discussion of the mechanism of calf diarrhea.

**Abstract:**

The complex etiology, higher morbidity and mortality, poor prognosis, and expensive cost of calf diarrhea have made it a catastrophic disease in the dairy industry. This study aims to assess the biomarkers in calves with diarrhea and to predict the biomarkers related to the pathway. As subjects, nine calves with diarrhea and nine healthy calves were enrolled, according to strict enrollment criteria. The serum metabolites were detected by a liquid chromatographic tandem mass spectrometry (LC-MS/MS), and then analyzed by online multivariate statistical analysis software to further screen the biomarkers. In addition, the biomarkers involved in the metabolic pathways of calves with diarrhea and healthy calves were analyzed. In the serum of calves with diarrhea, nine biomarkers were found to which several biomarkers exhibited a certain relation. Moreover, these biomarkers were involved in important metabolic pathways, including protein digestion and absorption, ABC transporters, aminoacyl-tRNA biosynthesis, mineral absorption, and fatty acid biosynthesis. All these findings suggested that the imbalance of these markers was closely related to the occurrence and development of calf diarrhea. The targeted regulation of metabolic pathways involved in these biomarkers may facilitate the diagnosis, treatment, and discussion of the mechanism of calf diarrhea.

## 1. Introduction

Calf diarrhea, a common gastrointestinal disease, causes great financial losses in the dairy industry due to its complex etiology, high morbidity and mortality, and poor prognosis [1,2,3,4]. The etiology and treatment of calf diarrhea have been the focus of researchers [5,6]. So far, it has been well documented that certain bacteria, viruses, parasites, feeding environments, and nutritional levels are all important causes of calf diarrhea [7,8]. The epidemiological investigation of calf diarrhea has shown that calf diarrhea is prevalent in almost all regions, and different pathogens cause the same clinical symptoms in calves, which is a huge challenge for clinical veterinarians in providing a timely diagnosis and treatment schedule [9,10]. Recently, calves with diarrhea were treated with antibiotics and prevented from catching the disease by improving the feeding conditions and vaccines [11,12,13]. However, the excessive use of broad-spectrum antibiotics causes bacterial resistance and drug residue problems. The isolation and identification of pathogens is the basis of targeted medicine, but pathogen diagnosis takes a certain amount of time and diagnostic equipment [3]. It takes 2–3 days to diagnose and start the treatment in a typical scenario on a dairy farm. It has attracted extensive attention in relation to the fight to reduce the pathogen diagnosis time by effectively alleviating clinical complications, including dehydration and acidosis.

It is essential to understand the body’s metabolism to effectively alleviate the clinical complications of calf diarrhea, because the clinical symptoms of many diseases are an external manifestation of the body’s metabolism imbalance [14,15]. To detect the changes in the substance metabolism of calves with diarrhea, several blood biochemical indicators have been assessed [16]. However, it is limited to looking at substance metabolism through several blood biochemical indicators. Metabolomics is an indispensable and readily used method for detecting changes in metabolism in response to various stimuli by analyzing alterations in endogenous small molecule metabolites [17,18]. It will be a promising approach to restore the metabolic balance and alleviate the clinical complications of diseases on the basis of metabolomics.

Calf diarrhea is manifested by the following symptoms: a thin water-like stool, cold ears and nose, throbbing bowels, oliguria, a pale or yellowish complexion, a smooth mouth, and a slow pulse. It is a common disorder in dairy farms. While calf diarrhea has a good conceptualization based on its clinical symptoms, the underlying mechanisms causing the clinical symptoms are still unclear, which makes it difficult to effectively alleviate the clinical complications associated with calves with diarrhea. In this study, the enrollment criteria of calves with diarrhea were drawn up on the basis of the clinical symptoms. The alterations in the metabolism balance of calves with diarrhea were investigated using metabolomics. On the basis of the biomarkers and their targeted metabolic pathways, the targeted regulation of the metabolic state of calves with diarrhea may contribute to the exploration of the molecular mechanism of calves with diarrhea and aid in the fight to reduce the pathogen diagnosis time.

## 2. Materials and Methods

### 2.1. Chemicals and Materials

Ammonium acetate (cat number: 70221) was supplied by Sigma (St. Louis, MO, USA). Deionized water (18.25 MW) was prepared with a Direct-Qfi3 system (Millipore, Bedford, MA, USA). MS-grade acetonitrile (cat number: 1499230-935) and MS-grade methanol (cat number: 103726) were purchased from Merck (Darmstadt, Germany). A vacuum blood collection tube was purchased from Laiwu Yaohua Pharmaceutical Packing Co., Ltd. (Shandong, China). A Sorvall Legend XT centrifuge was purchased from Thermo Fisher Scientific Inc. (Shanghai, China). An Agilent 1290 Infinity LC system was purchased from Agilent Technologies (Palo Alto, CA, USA). An AB Triple TOF 5600+ mass spectrometer was purchased from SCIEX (Foster City, CA, USA). An ACQUITY UPLC BEH Amide column was purchased from Waters (Milford, MA, USA).

### 2.2. Animals and Specimen Collection

This trial was conducted for two weeks at a dairy farm, located in the Gansu province in the northwest of China, housing approximately 5000 Holstein calves. Within 4 h after birth, all calves received 4 L of optimum quality colostrum by suckling or via an oro-esophageal feeder. The colostrum was harvested within 1 to 2 h after calving, and all calves were transferred to individual outdoor hutches within 24 h of birth. The calves with diarrhea were enrolled according to the following criteria: (1) fecal scores ≥ 3, according to the fecal scoring system [19], which is as follows: 1 = normal (formed), 2 = mild diarrhea (semi-formed pasty), 3 = moderate diarrhea (loose but stays on top of the bedding), and 4 = severe diarrhea (watery diarrhea that sifts through the bedding); (2) first occurrence of diarrhea, showing obvious clinical symptoms, such as cold ears and nose, throbbing bowels, oliguria, a pale or yellowish complexion, a smooth mouth, and a slow pulse, but not treated with drugs; (3) fourth parity, 10–14 days old and female. The calves enrolled as healthy calves (fecal scores = 1) were of the same sex, parity, and age as the calves with diarrhea and had a good mental and physical state and appetite. Besides, they did not show any clinical diarrhea symptoms. All enrolled calves had a clinical history of veterinary quarantine and clinical records. The protocols used in this study were in compliance with the Guidelines for the Care and Use of Laboratory Animals, as described by the US National Institutes of Health, and the protocols, including all sampling methods and experimental manipulations used in this study, were reviewed and approved by the Institutional Animal Care and Use Committee of the Lanzhou Institute of Husbandry and Pharmaceutical Science of CAAS (Animal Use Permit: SCXK201908–1259).

Blood samples of the calves were collected within 12 h of the first onset of diarrhea from the anterior vena cava. Moreover, all samples were collected before treatment of calf diarrhea. The blood was left at room temperature for 2 h and then centrifuged at 3500× *g* for 10 min at 4 °C, according to the technical specifications for the collection, storage, and transportation of veterinary diagnostic specimens of the People’s Republic of China Agricultural Industry Standard (NY/T541-2016). The serum was transferred to new cryopreservation tubes and stored in liquid nitrogen, prior to performing the metabolomics analysis.

### 2.3. Serum and Quality Control Sample Preparation

The serum samples were taken out of the liquid nitrogen and slowly thawed at 4 °C. From each sample, 20 μL of serum was taken, which was mixed with the rest to prepare a quality control (QC) sample. Then, the mixture was divided into aliquots, with the same volume as the other samples, and prepared using the method described below. A cold methanol/acetonitrile (1:1, v/v) mixture was added to the serum (4:1, v/v), vortexed for 1 min, incubated at −20 °C for 20 min, and centrifuged at 12,000× *g* for 15 min at 4 °C. The supernatant was transferred to a new tube and dried by a vacuum freeze dryer. The lyophilized powder was dissolved with 100 µL of acetonitrile/water (1:1, v/v), vortexed for 1 min, and then centrifuged at 14,000× *g* for 15 min at 4 °C. The supernatant was transferred into sample vials.

### 2.4. UPLC/QTOF-MS Analysis of Serum Metabolites

#### 2.4.1. Chromatographic Conditions

The serum metabolite analysis was performed with an Agilent 1290 Infinity LC system (Agilent Technologies, Palo Alto, CA, USA), coupled with a Triple TOF 5600+ mass spectrometer (AB/Sciex, Foster City, CA, USA). Chromatographic separation of serum samples was performed on an ACQUITY UPLC BEH Amide column (1.7 µm, 2.1 mm × 100 mm, Waters, Milford, MA, USA), which was maintained at 25 °C, and a 2 μL aliquot of each sample was injected into the column. The mobile phase consisted of solvent A (25 mM ammonium acetate and 25 mM ammonia in water) and B (acetonitrile). The optimized gradient program was established as shown in Table 1. The samples were randomly placed in a 4 °C autosampler throughout the analysis to avoid instrument detection signal fluctuations and to monitor and evaluate the system stability and reliability of the experimental data. To detect the stability of the instruments and systems, the QC sample was run at the beginning, the middle and the end of the sample queue.

#### 2.4.2. Mass Spectrometry Conditions

The serum metabolites were separated by UHPLC and analyzed by mass spectrometry using a Triple TOF 5600 mass spectrometer. Mass spectrometry was performed in both positive (ESI+) and negative (ESI−) electrospray ionization modes. The ESI source conditions were set as follows: ion source gas1 (Gas1), 60; ion source gas2 (Gas2), 60; curtain gas (CUR), 30; source temperature, 600 °C; and ion spray voltage floating (ISVF) ±5500 V (positive/negative modes). The mass spectrometry conditions were optimized as follows: TOF MS scan m/z range: 60–1000 Da; product ion scan m/z range: 25–1000 Da; TOF MS scan accumulation time: 0.20 s/spectra; and product ion scan accumulation time: 0.05 s/spectra. The MS/MS data were acquired using information-dependent acquisition (IDA) in high-sensitivity mode. The MS/MS conditions were set as follows: declustering potential: ±60 V; collision energy: 35 ± 15 eV; exclusion of isotopes: within 4 Da; candidate ions to monitor per cycle: 6.

### 2.5. Multivariate Statistical Analysis of Serum Metabolites Data

The raw MS data were initially converted into the mzXML format and imported into online multivariate statistical analysis software (XCMS) [20] to filter the noise, correct the baseline, align the peaks, and identity and quantify the peaks. Retention time errors of less than 0.1 min were applied to align the peaks. The obtained data were preprocessed using the SIMCA-P software (Umetrics AB, Umea, Sweden) through pattern recognition and Pareto-scaling, and then multivariate statistical analysis was performed, whereas principal component analysis (PCA), partial least squares discriminant analysis (OPLS-DA), and the evaluation of OPLS-DA models were performed on the dataset. The potential biomarkers were selected in accordance with the variable importance in projection (VIP) score > 1 from the OPLS-DA model. The potential biomarkers were further optimized by student’s t-test for the abundance of potential biomarkers of the calves with diarrhea and healthy calves. *p* < 0.05 was considered to be statistically significant. The biomarkers were further screened in accordance with a VIP score > 1, *p* < 0.05 and fold changes > 2 or <0.5.

### 2.6. Biomarkers Identification and Metabolic Pathways Analysis

The optimization of candidate biomarkers was performed by comparing the accuracy of the m/z values (<25 ppm), and the MS/MS spectra were interpreted using a self-built metabolite database (Shanghai Applied Protein Technology Co., LTD, shanghai, China), based on their MS and MS/MS signature. Cluster analysis and correlation analysis of the optimized candidate biomarkers were performed using R (version 3.6.1) [21]. The metabolic pathways involved in the optimized candidate biomarkers were identified using the KEGG database [22].

## 3. Results

### 3.1. Animal Enrollment and Quality Control of the Metabolites Detection System

The animals enrolled in this study were from isolated incidents. According to the enrollment criteria, 18 calves were included in the experiment: nine calves were placed in the healthy group, and the other nine were placed in the diarrhea group. The pathogen detection results showed that there were different types of pathogens in these calves with diarrhea (3 bacteria, 2 viruses, 1 parasite, 2 bacteria and virus, and 1 other). To assess the stability and reliability of the metabolites detection system, the alteration in the total ion chromatogram (TIC) and correlation based on the MS data among the QC samples were investigated. The results showed that the response intensity and retention time of the chromatographic peak of the QC samples were highly consistent (Figure S1). Moreover, the correlation coefficient of the MS data among the QC samples was more than 0.9 (Figure S1). All these results suggested that the metabolites detection system was stable and reliable.

### 3.2. The Alterations of the Metabolic Profile in the Serum of Diarrheal Calves

The serum metabolites of healthy calves and calves with diarrhea were detected using UPLC-Q-TOF/MS to explore the alteration of the metabolic profile. The representative total ion chromatograms (TICs) of the serum samples showed a fine separation and strong sensitivity of the established method. In the positive and negative ion modes, 3022 and 3397 metabolite ion peaks were identified, respectively.

To detect the metabolic profile of the healthy and diarrhea groups, principal component analysis (PCA) was performed, based on the MS data of the serum samples acquired in the positive and negative modes. The results showed that the parameter, R2X, of the PCA model was 0.586 and 0.603 in the positive and negative modes, respectively, suggesting that the obtained MS data were highly elucidated by the PCA models. The metabolic profiles of the serum samples from the healthy and diarrhea groups were clearly separated in the negative mode, while they could not be effectively separated in the positive mode. These findings suggested that, based on the generated PCA score plots, only data acquired in the negative mode had diagnostic values, since a clear distinction between the healthy and diarrhea groups was possible.

**Figure 1:**
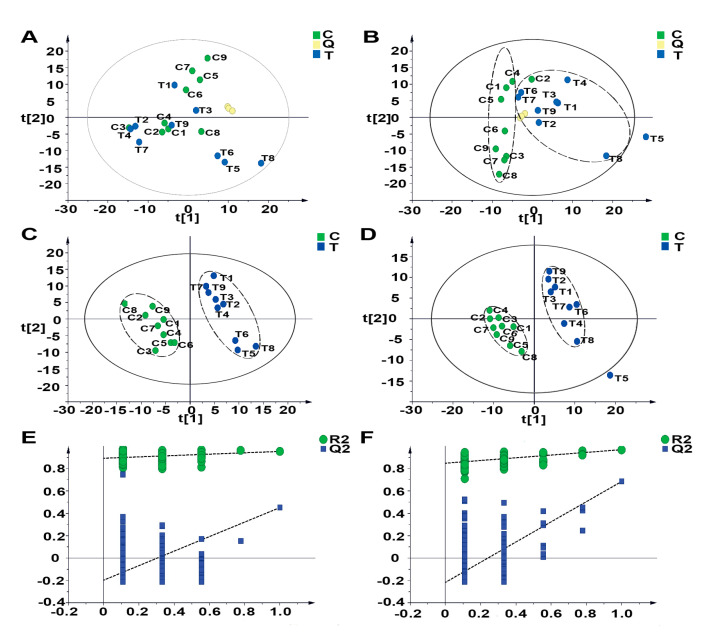
Differentiation of the metabolic profiles of the serum of healthy calves and that of calves with diarrhea. (**A**,**B**) PCA score plots based on the serum metabolic profiles of the healthy and diarrhea groups in the positive and negative modes: ESI+: R^2^ = 0.568, ESI−: R^2^ = 0.603. (**C**,**D**) OPLS-DA score plots of the healthy and diarrhea groups in the positive and negative modes: ESI+: R2X = 0.235, R2Y = 0.953, Q2 = 0.451; ESI−: R2X = 0.257, R2Y = 0.969, Q2 = 0.684. (**E**,**F**) Permutation test of the OPLS-DA model: ESI+: the intercepts of R^2^ = 0.892 and Q2 = −0.197, ESI−: the intercepts of R^2^ = 0.848 and Q2 = −0.217. C: healthy group; Q: quality control samples; T: diarrhea group.

To further investigate the potential biomarker of healthy and diarrheal calves, orthogonal projections to latent structures discriminate analysis (OPLS-DA) and a permutation test were performed, based on the obtained MS data. The permutation tests generated Q2 regression lines, with a negative intercept (Figure 1). In the positive and negative ionization modes, 329 and 388 potential biomarkers were screened in accordance with VIP > 1, respectively.

### 3.3. Serum Metabolome Differences between Healthy Calves and Calves with Diarrhea

**Figure 2:**
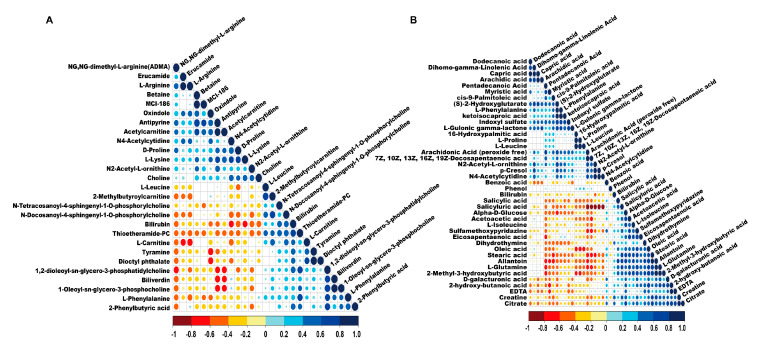
Correlation effects of potential biomarkers in the serum of calves with diarrhea in the positive (ESI+) electrospray ionization mode (**A**) and negative (ESI−) electrospray ionization mode (**B**), respectively. The circle dot scale represents the relevance of potential biomarkers.

**Figure 3:**
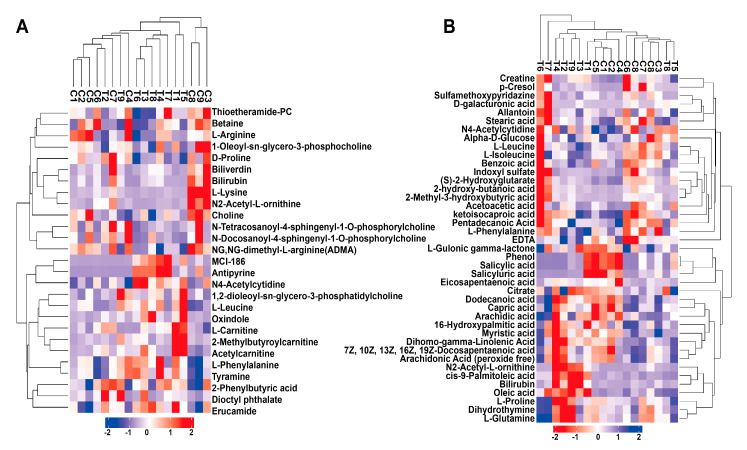
The relationship between healthy samples and diarrhea samples, and the expression patterns of the potential biomarkers in different samples in the positive (ESI+) electrospray ionization mode (**A**) and negative (ESI−) electrospray ionization mode (**B**). C: healthy group samples, T: diarrhea group samples.

In the positive and negative ionization modes, 27 and 41 potential biomarkers were found with a VIP score > 1 and *p* < 0.05, respectively. These potential biomarkers could be roughly divided into five categories, including amino acids, organic acids, fatty acids, amines, and other kinds. To clarify the relationship between the potential biomarkers, a correlation analysis of candidate biomarkers was performed. As indicated by the correlation analysis shown in Figure 2, many potential biomarkers had a strong positive or negative correlation with each other. Among amino acids metabolites, proline was positively correlated with L-leucine and L-lysine. Dodecanoic acid, a kind of fatty acids metabolite, was positively correlated with capric acid and arachidic acid. These findings suggested that the closely related potential biomarkers might be involved in the same or related metabolic pathway. To assess the rationality of a potential candidate, we comprehensively and intuitively found the differential abundance patterns of potential biomarkers among the different serum samples by performing cluster analysis. The dendrogram coupled with a heat map showed that most serum samples of calves with diarrhea formed a cluster and were separated from the serum samples of healthy calves based on the abundance of potential biomarkers in the negative (ESI−) electrospray ionization mode, while in the positive (ESI+) electrospray ionization mode, the serum samples of the calves with diarrhea and healthy calves could not be clustered well (Figure 3), which was similar to the results of OPLS-DA analysis. These results suggested that the potential biomarkers screened in the negative (ESI−) electrospray ionization mode were better interpretable for experiment subjects, compared to candidate biomarkers screened in the positive (ESI+) electrospray ionization mode.

**Figure 4:**
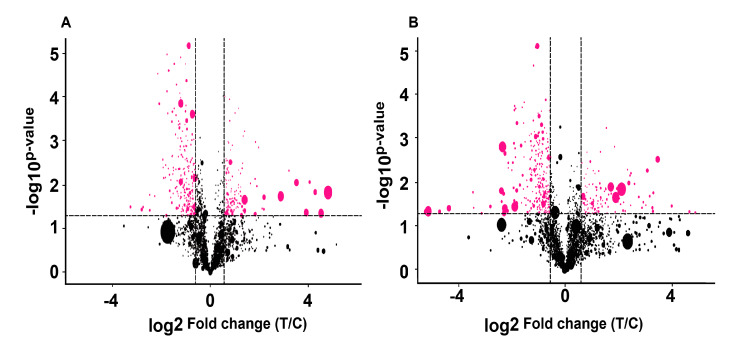
The candidate biomarkers in the serum of healthy calves and calves with diarrhea. The circle dot scale represents the variable importance of the projection (VIP) value; the red circle dot represents the metabolites that had a *p*-value < 0.05 and fold change > 1.5. (**A**) Positive (ESI+) electrospray ionization mode; (**B**) Negative (ESI−) electrospray ionization mode.

The potential biomarkers were further optimized, with a VIP score > 1, *p* < 0.05 and fold changes > 1.5 in positive and negative ionization (Figure 4). In the positive and negative ionization modes, 16 and 27 candidate biomarkers were found, respectively. Moreover, antipyrine was identified as a candidate biomarker in the serum of healthy calves and calves with diarrhea. Antipyrine, an antipyretic analgesic and anti-inflammatory drug, could be used to relieve postpartum pain in dairy cows. We speculate that the antipyrine might be from colostrum. In addition, nine biomarkers were found with a VIP > 1, *p* < 0.05 and fold changes > 2 or <0.5, as shown in Table 2.

### 3.4. The Disorder in the Metabolic Pathways of Calves with Diarrhea

**Figure 5:**
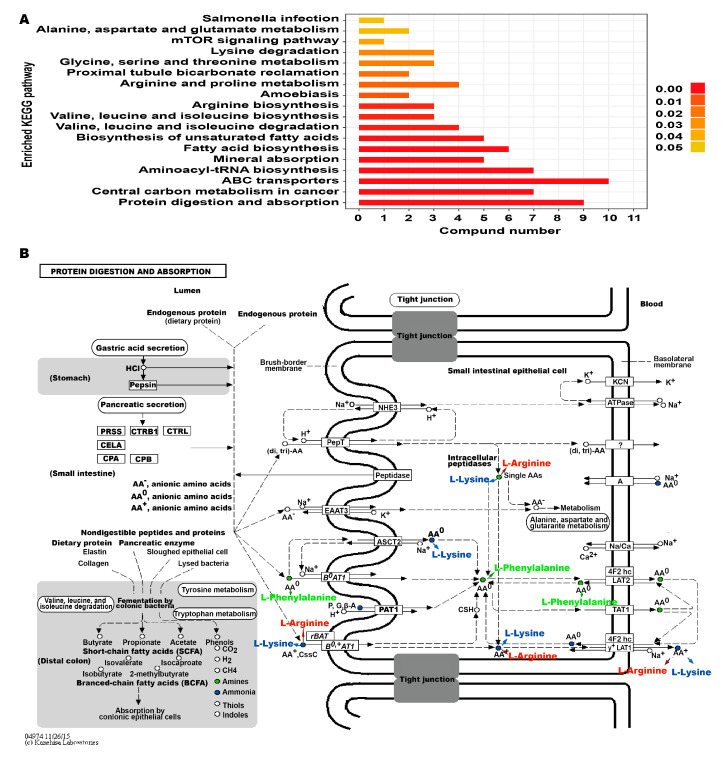
The enriched KEGG pathway (**A**) as well as protein digestion and absorption pathway involving the candidate biomarkers (**B**).

To investigate the effect of the candidate biomarkers on metabolic pathways, KEGG pathway enrichment analysis was performed using the KEGG database. As indicated by the KEGG pathway enrichment analysis and KEGG pathway shown in Figure 5, protein digestion and absorption, ABC transporters, aminoacyl-tRNA biosynthesis, and the mineral absorption pathway were significantly changed in calves with diarrhea. Nine candidate biomarkers including L-arginine and L-lysine were involved in the protein digestion and absorption pathway. Ten candidate biomarkers were related to the ABC transporters pathway. Moreover, several candidate biomarkers including arginine, L-lysine, L-leucine and L-isoleucine, were simultaneously enriched into multiple metabolic pathways. These findings suggested that the balance of mineral absorption, protein digestion and absorption, and ABC transporters might be disturbed in calves with diarrhea, which caused the significant changes of candidate biomarkers.

## 4. Discussion

Diarrhea, a common gastrointestinal disease, is widely prevalent in dairy farming [7]. The immature immune system and poor self-regulation ability of calves make them the main subjects of diarrheal diseases [23,24]. To find a targeted treatment for and explore the molecular mechanism of calves with diarrhea, many studies focus on the development of pathogen identification technology and exploration of the pathogenic mechanism. The complex pathogen and urgent course of calf diarrhea constitutes a huge challenge for pathogen identification and studies on pathogenic mechanism in calves with diarrhea. Metabolomics can quickly, sensitively, and comprehensively detect changes in the metabolites of the organism under physiological or pathological conditions, which contributes to the identification of diagnostic markers and investigation of the pathogenic mechanism in calves with diarrhea [25,26]. In the present study, in order to avoid the effects of parity, age, gender, and other factors on the metabolites of the organism, 18 calves were enrolled in the project, according to the strict enrollment criteria. The metabolites in the serum were detected by UPLC-Q-TOF/MS. The results of the principal component analysis, based on the acquired metabolites data, suggested that there were significant changes in the metabolites of healthy calves and calves with diarrhea. However, in the diarrhea group, two samples could not be clustered in the cluster analysis, based on the candidate biomarkers screened in the negative (ESI−) electrospray ionization mode. The complexity of the clinical samples and the complex biological information of clinical samples could not be completely represented and clarified by several special biomarkers. In the present study, most calves with diarrhea were clustered into a cluster based on candidate biomarkers, which suggested that these biomarkers might play a vital role in calf diarrhea.

To further investigate the biological significance reflected by these candidate biomarkers, the candidate biomarkers and the signal pathway involved in the candidate biomarkers were identified by MS/MS, combined with the related database, such as the self-built metabolite database and KEGG database. The candidate biomarkers, including indoxyl sulfate, *p*-cresol, 2-methyl-3-hydroxybutyric acid, D-galacturonic acid, 2-hydroxy-butanoic acid, and acetoacetic acid, were significantly increased in the serum of calves with diarrhea. We speculate that the antipyrine may be from colostrum. As for the physiological condition, indoxyl sulfate is a metabolite of dietary protein or tryptophan, while an excessive accumulation of indoxyl sulfate would cause physiological disorders, such as an increase of reactive oxygen species (ROS) and a decrease of antioxidant capacity [27,28]. Moreover, it has been documented that indoxyl sulfate is also a microbial metabolite of *Escherichia* [29]. In the present study, we speculated that the increase of indoxyl sulfate might be associated with the intestinal *Escherichia* imbalance in calves with diarrhea. A previous study of our group also showed that *Escherichia* is a major pathogen in calves with diarrhea. In server gastrointestinal disorders, alterations in the intestinal flora could cause an overgrowth of specific bacteria that are *p*-cresol producers [30,31]. The increase of *p*-cresol might be associated with the intestinal flora of calves with diarrhea. The immoderate *p*-cresol would block cell K+ channels, resulting in an electrolyte imbalance [32]. The increase of 2-methyl-3-hydroxybutyric acid, 2-hydroxy-butanoic acid, and acetoacetic acid in the serum of calves with diarrhea might be the result of a metabolic imbalance of ketogenic amino acid and ketone body.

The results of the signal pathway involved in the candidate biomarkers suggested that the protein digestion and absorption, ABC transporter, mineral absorption pathway, fatty acid and unsaturated fatty acid biosynthesis, and aminoacyl-tRNA biosynthesis pathway in calves with diarrhea changed significantly. It has been well documented that an imbalance of protein digestion and absorption and mineral absorption plays a pivotal role in diarrhea. On the basis of the biomarkers and their targeted metabolic pathways, the targeted regulation of the metabolic state of calves with diarrhea may contribute to the exploration of the molecular mechanism of calves with diarrhea.

Metabolomics is an effective and irreplaceable method for finding biomarkers in many diseases, but the quality and credibility of the biomarkers is dependent on large samples. Therefore, more samples need to be enrolled to clarify the biological significance of the biomarkers.

## 5. Conclusions

The metabolic profile of calves with diarrhea had undergone significant changes compared with that of healthy calves. Moreover, the occurrence and development of calf diarrhea were closely related to the changes of nine significant biomarkers and their targeted metabolic pathways. The targeted regulation in the metabolic state of calves with diarrhea may have great potential to facilitate the diagnosis, treatment, and discussion about the mechanism of calf diarrhea.

## Figures and Tables

**Table 1:** Optical UPLC gradient elution program of the serum samples.

Time (min)	A%	B%	Column Temperature (°C)	Flow Rate (mL/min)
0	5.0	95.0	25	0.3
1	5.0	95.0	25	0.3
14	35.0	65.0	25	0.3
16	60.0	40.0	25	0.3
18	60.0	40.0	25	0.3
18.1	5.0	95.0	25	0.3
23	5.0	95.0	25	0.3

**Table 2:** The result of biomarkers identified in the serum of calves with diarrhea.

Metabolite	VIP	*p-*Vaule	Fold Change (T/C)	Retention Time (min)	SM
Oxindole	1.4	0.017	7.28	0.53	+
Acetylcarnitine	7.5	0.021	2.66	10.04	+
L-lysine	1.8	0.032	0.1	14.55	+
N2-acetyl-L-ornithine	1.3	0.032	0.15	11.94	+
2-methylbutyroylcarnitine	3.2	0.046	3.6	7.95	+
(S)-2-Hydroxyglutarate	1.3	0.01	2.44	11.17	−
Indoxyl sulfate	10.7	0.014	4.29	4.64	−
*p*-Cresol	1	0.029	2.82	8.15	−
Benzoic acid	3	0.029	2.01	2.75	−

RT: retention time; VIP: variable importance in the projection; SM: scan mode; +: metabolites identified in the positive (ESI+) electrospray ionization mode; −: metabolites identified in the negative (ESI−) electrospray ionization mode. T/C: calves with diarrhea, compared with healthy calves.

## Supplementary Materials

The following are available online at https://www.mdpi.com/2076-2615/10/5/769/s1, Figure S1: Ion chromatogram and correlation analysis of QC samples.

## References

[1] Cho Y.I., Yoon K.J. (2014). An overview of calf diarrhea—infectious etiology, diagnosis, and intervention. J. Vet. Sci..

[2] Mohammed S.A.E., Marouf S.A.E., Erfana A.M., El-Jakee J., Hessain A.M., Dawoud T.M., Kabli S.A., Moussa I.M. (2019). Risk factors associated with E. coli causing neonatal calf diarrhea. Saudi J. Biol. Sci..

[3] Smith D.R. (2012). Field disease diagnostic investigation of neonatal calf diarrhea. Vet. Clin. North Am. Food Anim. Pract..

[4] Reiten M., Rousing T., Thomsen P.T., Otten N.D., Forkman B., Houe H., Sorensen J.T., Kirchner M.K. (2018). Mortality, diarrhea and respiratory disease in Danish dairy heifer calves: Effect of production system and season. Prev. Vet. Med..

[5] Bonelli F., Turini L., Sarri G., Serra A., Buccioni A., Mele M. (2018). Oral administration of chestnut tannins to reduce the duration of neonatal calf diarrhea. BMC Vet. Res..

[6] Mercado R., Pena S., Ozaki L.S., Fredes F., Godoy J. (2015). Multiple Cryptosporidium parvum subtypes detected in a unique isolate of a Chilean neonatal calf with diarrhea. Parasitol. Res..

[7] Cruvinel L.B., Ayres H., Zapa D.M.B., Nicaretta J.E., Couto L.F.M., Heller L.M., Bastos T.S.A., Cruz B.C., Soares V.E., Teixeira W.F. (2019). Prevalence and risk factors for agents causing diarrhea (*Coronavirus*, *Rotavirus*, *Cryptosporidium* spp., *Eimeria* spp., and nematodes helminthes) according to age in dairy calves from Brazil. Trop. Anim. Health Prod..

[8] Peter S.G., Gitau G.K., Richards S., Vanleeuwen J.A., Uehlinger F., Mulei C.M., Kibet R.R. (2016). Risk factors associated with *Cryptosporidia*, *Eimeria*, and diarrhea in smallholder dairy farms in Mukurwe-ini Sub-County, Nyeri County, Kenya. Vet. World.

[9] Blanchard P.C. (2012). Diagnostics of dairy and beef cattle diarrhea. Vet. Clin. North Am. Food Anim. Pract..

[10] Klein-Jobstl D., Iwersen M., Drillich M. (2014). Farm characteristics and calf management practices on dairy farms with and without diarrhea: A case-control study to investigate risk factors for calf diarrhea. J. Dairy Sci..

[11] Roussel A.J., Brumbaugh G.W. (1991). Treatment of diarrhea of neonatal calves. Vet. Clin. North Am. Food Anim. Pract..

[12] Renaud D.L., Kelton D.F., Weese J.S., Noble C., Duffield T.F. (2019). Evaluation of a multispecies probiotic as a supportive treatment for diarrhea in dairy calves: A randomized clinical trial. J. Dairy Sci..

[13] Fortuoso B.F., Volpato A., Rampazzo L., Glombowsky P., Griss L.G., Galli G.M., Stefani L.M., Baldissera M.D., Ferreira E.B., Machado G. (2018). Homeopathic treatment as an alternative prophylactic to minimize bacterial infection and prevent neonatal diarrhea in calves. Microb. Pathog..

[14] Mamas M., Dunn W.B., Neyses L., Goodacre R. (2011). The role of metabolites and metabolomics in clinically applicable biomarkers of disease. Arch. Toxicol..

[15] Yan M., Xu G. (2018). Current and future perspectives of functional metabolomics in disease studies—A review. Anal. Chim. Acta.

[16] Gultekin M., Voyvoda H., Ural K., Erdogan H., Balikci C., Gultekin G. (2019). Plasma citrulline, arginine, nitric oxide, and blood ammonia levels in neonatal calves with acute diarrhea. J. Vet. Intern. Med..

[17] Noorbakhsh H., Yavarmanesh M., Mortazavi S.A., Adibi P., Moazzami A.A. (2019). Metabolomics analysis revealed metabolic changes in patients with diarrhea-predominant irritable bowel syndrome and metabolic responses to a synbiotic yogurt intervention. Eur. J. Nutr..

[18] Alghamdi A., Gerasimidis K., Blackburn G., Akinci D., Edwards C., Russell R.K., Watson D.G. (2018). Untargeted Metabolomics of Extracts from Faecal Samples Demonstrates Distinct Differences between Paediatric Crohn’s Disease Patients and Healthy Controls but No Significant Changes Resulting from Exclusive Enteral Nutrition Treatment. Metabolites.

[19] Meganck V., Hoflack G., Piepers S., Opsomer G. (2015). Evaluation of a protocol to reduce the incidence of neonatal calf diarrhoea on dairy herds. Prev. Vet. Med..

[20] Benton H.P., Ivanisevic J., Mahieu N.G., Kurczy M.E., Johnson C.H., Franco L., Rinehart D., Valentine E., Gowda H., Ubhi B.K. (2015). Autonomous metabolomics for rapid metabolite identification in global profiling. Anal. Chem..

[21] Daróczi G. (2015). Mastering Data Analysis with R.

[22] Kanehisa M., Goto S., Sato Y., Furumichi M., Tanabe M. (2012). KEGG for integration and interpretation of large-scale molecular data sets. Nucleic Acids Res..

[23] Bok M., Alassia M., Frank F., Vega C.G., Wigdorovitz A., Parreno V. (2018). Passive immunity to control Bovine coronavirus diarrhea in a dairy herd in Argentina. Rev. Argent. De Microbiol..

[24] Johnson K.F., Chancellor N., Burn C.C., Wathes D.C. (2017). Prospective cohort study to assess rates of contagious disease in pre-weaned UK dairy heifers: Management practices, passive transfer of immunity and associated calf health. Vet. Rec. Open.

[25] Kojouri G.A., Hassanpour H., Taghavi N., Taghadosi C. (2012). Nitric oxide metabolites status in calves with acute and chronic diarrhea. Comp. Clin. Pathol..

[26] Basoglu A., Sen I., Meoni G., Tenori L., Naseri A. (2018). NMR-Based Plasma Metabolomics at Set Intervals in Newborn Dairy Calves with Severe Sepsis. Mediat. Inflamm..

[27] Duranton F., Cohen G., De Smet R., Rodriguez M., Jankowski J., Vanholder R., Argiles A., European Uremic Toxin Work Group (2012). Normal and pathologic concentrations of uremic toxins. J. Am. Soc. Nephrol..

[28] Dou L., Jourde-Chiche N., Faure V., Cerini C., Berland Y., Dignat-George F., Brunet P. (2007). The uremic solute indoxyl sulfate induces oxidative stress in endothelial cells. J. Thromb. Haemost..

[29] Evenepoel P., Meijers B.K., Bammens B.R., Verbeke K. (2009). Uremic toxins originating from colonic microbial metabolism. Kidney Int..

[30] Passmore I.J., Letertre M.P.M., Preston M.D., Bianconi I., Harrison M.A., Nasher F., Kaur H., Hong H.A., Baines S.D., Cutting S.M. (2018). Para-cresol production by Clostridium difficile affects microbial diversity and membrane integrity of Gram-negative bacteria. PLoS Pathog..

[31] Sivsammye G., Sims H.V. (1990). Presumptive identification of Clostridium difficile by detection of p-cresol in prepared peptone yeast glucose broth supplemented with p-hydroxyphenylacetic acid. J. Clin. Microbiol..

[32] Vanholder R., De Smet R., Lesaffer G. (1999). p-cresol: A toxin revealing many neglected but relevant aspects of uraemic toxicity. Nephrol. Dial. Transplant. Off. Publ. Eur. Dial. Transpl. Assoc. Eur. Ren. Assoc..

