# Supplementary figures and images for: Serum Metabolomics Revealed the Differential Metabolic Pathway in Calves with Severe Clinical Diarrhea Symptoms
Source: Animals (Basel). 2020 Apr 28;10(5):769. doi: 10.3390/ani10050769 (PMC7278412; doi:10.3390/ani10050769)

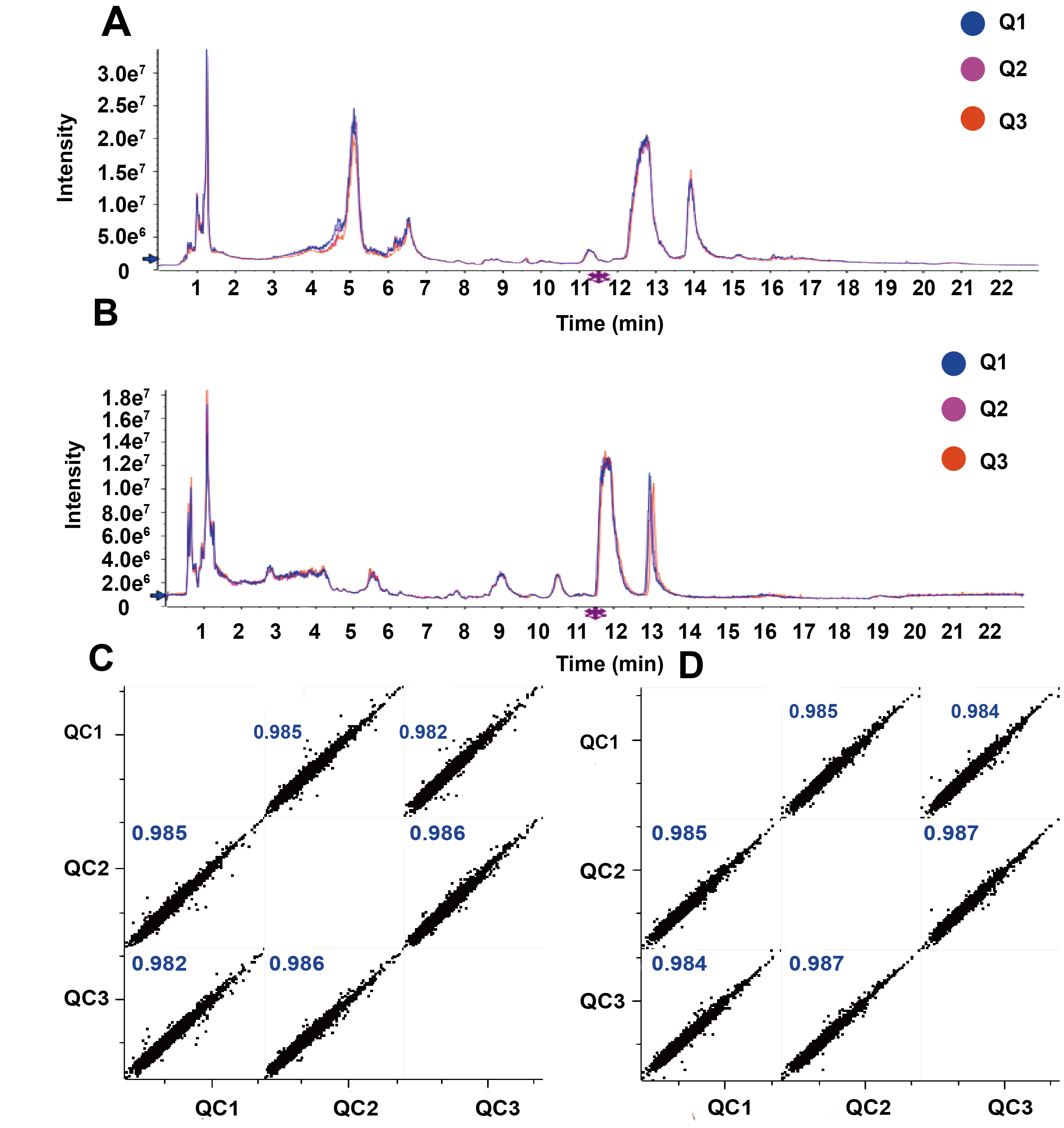

Supplement: Supplementary file 1 [file animals-10-00769-s001.tif]
